# Monitoring the Transmission of *Schistosoma japonicum* in Potential Risk Regions of China, 2008 – 2012

**DOI:** 10.3390/ijerph110202278

**Published:** 2014-02-21

**Authors:** Hui Dang, Jing Xu, Shi-Zhu Li, Zhi-Guo Cao, Yi-Xin Huang, Cheng-Guo Wu, Zu-Wu Tu, Xiao-Nong Zhou

**Affiliations:** 1 National Institute of Parasitic Diseases, Chinese Center for Disease Control and Prevention, Laboratory of Parasite and Vector Biology, Ministry of Public Health, WHO Collaborating Centre for Malaria, Schistosomiasis and Filariasis, Shanghai 200025, China; E-Mails: danghuinew@hotmail.com (H.D.); xfmjing@163.com (J.X.); lisz@chinacdc.cn (S.-Z.L.); 2 Anhui Institute of Schistosomiasis Control, Hefei 230061, China; E-Mail: ahzhiguo@126.com; 3 Jiangsu Institute of Schistosomiasis Control, Wuxi 214064, China; E-Mail: huang_yixin@163.com; 4 Chongqing Center for Disease Control and Prevention, Chongqing 400042, China; E-Mail: wcguo94@163.com; 5 Hubei Institute of Schistosomiasis Control, Wuhan 430079, China; E-Mail: tuzuwu@163.com

**Keywords:** Schistosomiasis, *Oncomelania hupensis*, surveillance, potential endemic areas

## Abstract

Schistosomiasis japonica, caused by *Schistosoma japonicum* infection, remains a major public health concern in China, and the geographical distribution of this neglected tropical disease is limited to regions where *Oncomelania hupensis*, the intermediate host of the causative parasite, is detected. The purpose of this study was to monitor the transmission of *S. japonicum* in potential risk regions of China during the period from 2008 through 2012. To monitor the transmission, 10 fixed surveillance sites and 30 mobile sentinel sites were selected in 10 counties of four provinces, namely Anhui, Jiangsu, Chongqing and Hubei. There were 8, 9, 6, 2 and 3 cases infected with *S. japonicum* detected in the 30 mobile sentinel sites during the 5-year study period, while 27 subjects were positive for the antibody-based serum test in the 10 fixed sentinel sites; however, no infection was found. In addition, neither local nor imported livestock were found to be infected. No *O. hupensis* snails were detected in either the fixed surveillance or the mobile sentinel sites; however, the snail host was found to survive and reproduce at Chaohu Lake, inferring the potential of transmission of the disease. It is suggested that the continuous surveillance of schistosomiasis japonica should be carried out in both the endemic foci and potential risk regions of China, and an active, sensitive system to respond the potential risk of transmission seems justified.

## 1. Introduction

Schistosomiasis japonica, which is a snail-transmitted, water-borne devastating neglected tropical disease caused by infection of *Schistosoma japonicum*, remains a major public health concern in China [1,2,3], and the distribution of the disease is governed by the intermediate host *Oncomelania hupensis* [4,5]. Currently, the transmission of *S. japonicum* is mainly concentrated in five provinces along the middle and lower reaches of the Yangtze River and some mountainous regions of the Yunnan and Sichuan provinces, and over 0.7 million people are thought to be infected with the parasite in China [6]. 

The impact of water resource development and global climate change on the transmission of schistosomiasis has been illustrated [7,8,9,10,11,12]. It is predicted that the global warming would cause the expansion of the current snail habitants northward, resulting in the potential of transmission of schistosomiasis japonica in non-endemic regions, north of China [13]. The water resource development including the South-to-North Water Diversion Project, the Yangtze-to-Chaohu Water Diversion Project, the Yangtze-to-Hanjiang Water Diversion Project and the Three Gorges Dam all cross the snail-breeding regions, and the effect of the construction of these projects on the original landscape of schistosomiasis transmission has been paid much attention [11,14,15,16]. 

The present study was designed to monitor the transmission of *S. japonicum* in 10 fixed surveillance sites and 30 mobile sentinel sites from 10 counties of Anhui, Jiangsu, Chongqing and Hubei provinces, China, on the water diversion route of the projects where a potential of transmission of schistosomiasis japonica is defined during the period from 2008 to 2012, so as to provide the data support for the formulation of the control strategy of schistosomiasis in potential risk regions and the establishment of a surveillance-response system. 

## 2. Methods

### 2.1. Study Areas

**Figure 1:**
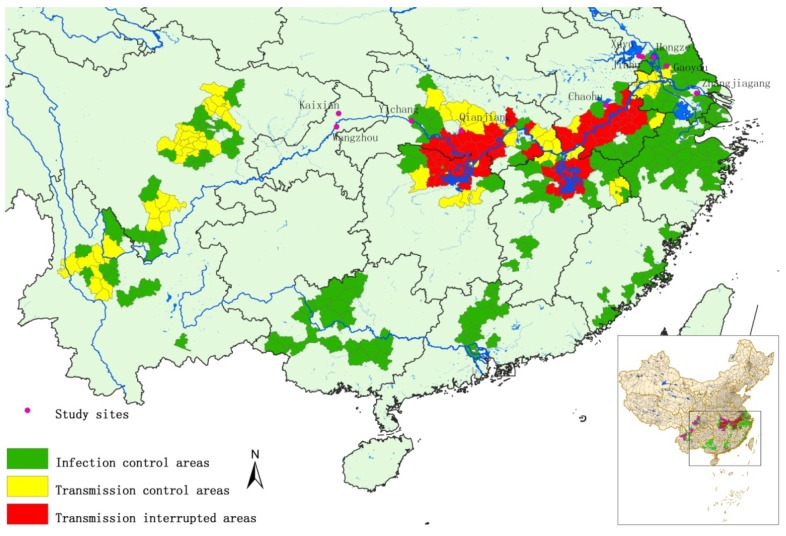
The distribution of surveillance sites for potential schistosomiasis japonica endemic areas in P.R. China.

A total of 10 counties of the four provinces, namely Anhui, Jiangsu, Chongqing and Hubei, were involved in the study, including (i) Gaoyou, Hongze, Jinhu, Xuyu and Zhangjiagang counties of Jiangsu province located along the water-diversion route of Eastern Route Project of South-to-North Water Diversion Project, (ii) Chaohu county of Anhui province neighboring the water-diversion route of Yangtze-to-Chaohu Water Diversion Project, (iii) Qianjiang county of Hubei province distributed along the water-diversion route of Yangtze-to-Hanjiang Water Diversion Project, (iv) Yichang county of Hubei province downstream of the Three Gorges Dam, (v) Wanzhou and Kaixian counties of Chongqing located in the reservoir areas of Three Gorge Dam. All counties selected are located adjacent to the schistosomiasis endemic regions or connecting with the endemic foci, and all these regions are non-endemic for *S. japonicum* historically. The water-resource development results in a high potential for introduction of *O. hupensis*, which is therefore defined as potential risk regions. A fixed surveillance site and 3 mobile sentinel sites were assigned in each county, consequently 10 fixed sites and 30 mobile sites designed for the monitoring of the study (Figure 1).

### 2.2. Monitoring S. japonicum Infection in Humans and Livestock

In each surveillance or sentinel site, 300–500 local residents at age of 6–65 years sampled were surveyed for *S. japonicum* infection by indirect haemagglutination assay (IHA) [17], followed by Kato-Katz method (three slides from a single stool sample) [18]. The floating populations who lived in the surveillance or sentinel site over one month were also surveyed using the aforementioned technique. In addition, the local livestock and those purchased from the schistosomiasis endemic areas were surveyed for *S. japonicum* infection using the miracidium-hatching test (MHT) (three slides from a single stool sample) in each fixed surveillance site and mobile sentinel site [19]. The positive individuals found were treated with praziquantel at a single oral dose of 40 mg/kg. 

### 2.3. Snail Surveillance

Snail survey was performed in three suspected settings (with an area of less than 1 km^2^) that connect with lakes or rivers at each fixed surveillance site by using systematic sampling in combination with environmental sampling at spring during the study period, whereas five suspected settings sampled from each mobile sentinel site were investigated by using the environmental sampling technique. In addition, those materials suspected of carrying snails, including floating debris, boats and water plants were examined. All snails captured were dissected and examined for death and *S. japonicum* infection under a microscope.

### 2.4. Monitoring Snail Survival and Reproduction

Sixty active, adult imported *O. hupensis* snails, with a male:female ratio of approximately 1, were caged in two marshlands located in Zhangjiagang County of Jiangsu Province and Chaohu County of Anhui Province, while those raised in the marshland of Wuwei County, Anhui Province, a historically endemic focus, served as control. Cages were removed from the field and snails in these cages were recovered. Those suspected of being dead were tested by using the knocking method, and the live adult, juvenile snails and snail eggs were counted. 

### 2.5. Statistical Analysis

All data were entered in Excel (Microsoft Corporation; Redmond, WA, USA) and all statistical analyses were performed using the statistical software Statistical Package for the Social Sciences v. 11.0 (SPSS 11.0, SPSS Inc., Chicago, IL, USA). Differences of proportions were tested for statistical significance with the chi-square test. A *p* value < 0.05 was considered statistically significant. 

## 3. Results

### 3.1. S. japonicum Infection in Humans and Livestock

**Figure 2:**
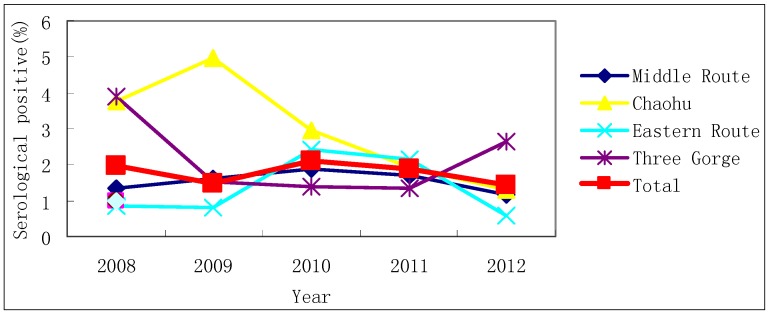
Results obtained from examination of the floating population in potential endemic areas, from 2008 to 2012.

Of the 7,437 and 4,939 local residents screened for *S. japonicum* infection using IHA in the 10 fixed surveillance sites and 30 mobile sentinel sites in 2008 and 2012, 58 and 25 subjects were sero-positive (Table 1), however, no infection was detected in any of the sero-positive cases by the Kato-Katz technique. During the study period from 2008 to 2012, the sero-prevalence of *S. japonicum* infection was 1.96%, 1.50%, 2.10%, 1.88% and 1.43% in the floating population, respectively (Figure 2), and 8, 9, 6, 2 and 3 cases were found to be infected with *S. japonicum*. A total of 878 local livestock and 879 imported livestock were examined from 2008 to 2012 in four counties of Qianjiang, Chaohu, Gaoyou and Wanzhou, but no infection was found. 

**Table 1:** Seroprevalence of human schistosomiasis in the fixed population in potential endemic areas, 2008 and 2012.

Province	County	Serological Test, 2008			Serological Test, 2012		
		No. detected	No. positive	Positive rate (%)	No. detected	No. positive	Positive rate (%)
Anhui	Chaohu	301	0	0	314	0	0
Chongqing	Wanzhou	501	5	1	501	2	0.4
	Kaixian	500	18	3.6	300	4	1.33
Hubei	Qianjiang	2,275	26	1.14	313	3	0.96
	Yichang	101	0	0	303	1	0.33
Jiangsu	Xuyu	1,035	6	0.58	638	5	0.78
	Hongze	514	3	0.58	555	5	0.9
	Jinhu	1,539	0	0	1,021	5	0.49
	Gaoyou	671	0	0	494	0	0
	Zhangjianggang	-	-	-	500	0	0
Total		7,437	58	0.78	4,939	25	0.51

### 3.2. Snail Status

During the study period from 2008 to 2012, 38, 38, 44, 41 and 36 snail habitats, with a total area of 682.64 ha, were surveyed, respectively; however, no snails were detected in either the fixed surveillance sites or the mobile sentinel sites. In eight counties of Chaohu, Yichang, Hongze, Jinhu, Xuyu, Zhangjiagang, Wanzhou and Kaixian, a total of 28,285.80 kg floating debris were recovered, but no *O. hupensis* snails were found. 

### 3.3. Snail Survival and Reproduction

**Figure 3:**
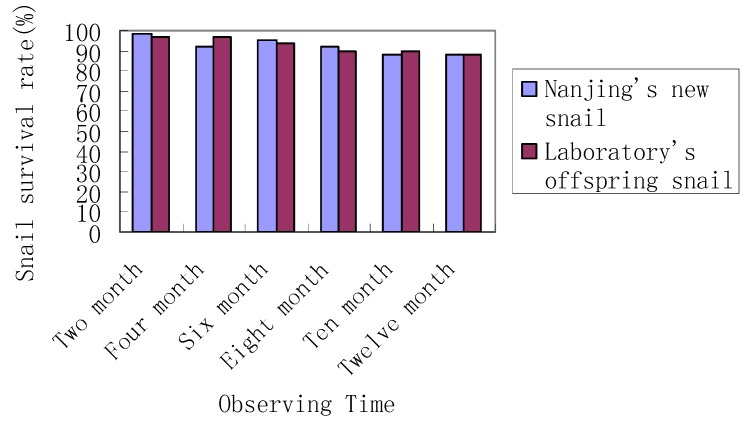
Observed snail survive rate at Jiangsu’s Zhangjiagang County.

**Figure 4:**
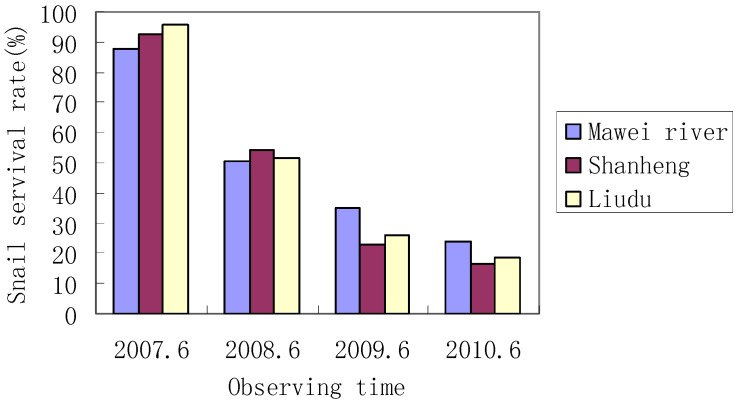
Observed snail survive rate at Anhui’s Chaohu County.

The one-year survival rate of the imported *O. hupensis* snails raised in the laboratory and the marshland of Zhangjiagang County was both over 88%, and no statistically significant difference was observed between the survival of snails raised in the laboratory and the field (*p* > 0.05; Figure 3). The survival of snails caged in Chaohu and Wuwei counties reduced year by year during the period from 2007 through 2010, and no significant difference was found (*p* > 0.05; Figure 4).

In Zhangjiagang, the number of imported snails increased from 60 and 60 to 1,610 and 1,620 during one year, respectively, and a pair of snails were found to produce 40 offspring snails after one year (Table 2). In Chaohu, three snail group reproduced. The number of the three groups of snails increased from 100, 100 and 100 to 396, 374 and 412, respectively from June 2007 to June 2010, and *O. hupensis* snails were found to survive, reproduce and produce offspring snails at Chaohu Lake under the laboratory conditions (Table 3). 

**Table 2:** Reproduction of offspring snails in Zhangjiagang, Jiangsu Province.

Snail population	April	June	August	October	December	Next February
Nanjing field-derived snails	60	60	60	800	1,334	1,610
Laboratory offspring snails	60	60	60	911	1,326	1,620

**Table 3:** Reproduction of offspring snails in Chaohu Lake, Anhui Province.

Site	2007–2006	2008–2006	2009–2006	2010–2006
Mawei River	100	272	411	396
Shanheng	100	256	346	374
Liudu	100	289	387	412

## 4. Discussion

It has been reported that the water resource development may result in the intensification of schistosomiasis transmission or introduce schistosomiasis into non-endemic areas. Transmission of *S. mansoni* and *S. haematobium*, for example, has been associated with the construction of large dams in several African countries, such as Egypt, Ghana, Niger, Nigeria, Tanzania, Cameroon, Sudan, and Senegal [20,21,22,23]. Since *Biomphalaria* and *Bulinus* snails, the intermediate hosts of *S. mansoni* and *S. haematobium*, are endemic on the African mainland, relatively eurythermal and entirely aquatic, it is easy to see how they may be spread by the water flows associated with irrigation projects and so colonise new areas. *O. hupensis*, the only endemic intermediate host of *S. japonicum* in China, is slightly different, being both stenothermal and amphibious [24]. Schistosomiasis is considered a sensitive indicator of ecological alterations due to its wide distribution and rapid change in morbidity. In a systematic literature review and meta-analysis with aims to quantify the risk of water resources development and management on schistosomiasis, it was concluded that the development and management of water resources is an important risk factor for schistosomiasis, and strategies to mitigate negative effects should be integrated into the planning, implementation, and operation of future water projects [25]. 

It has been shown that the South-to-North Water Diversion Project may result in the northward spread of schistosomiasis japonica in the context of a significant rise in the minimum winter temperatures in northern China caused by global warming. In addition, an expansion of schistosomiasis transmission into currently non-endemic areas in the north has been predicted, with an additional risk area of 783,883 km^2^ resulting from a rise of 1.6 degrees C by 2050, translating to 8.1% of the surface area of China [26]. A systematic review has revealed that the Three Gorges Dam is capable of inducing a wide variety of environmental and ecological changes, both within the Three Gorges region and in downstream areas. These changes, however, carry ambivalent implications for the reproduction of *Oncomelania* snails and the spreading of schistosome infections [10]. Furthermore, major changes in the demographics and agricultural practices of the Three Gorges and downstream Yangtze areas caused by the dam could also exert significant influence on the transmission of schistosomiasis in these regions [11]. In addition, there are many studies reporting the impact of the Yangtze-to-Chaohu Water Diversion Project and the Yangtze-to-Hanjiang Water Diversion Project on the transmission of schistosomiasis japonica [15,16]. However, whether the construction of the water resource development affects schistosomiasis transmission in potential risk regions of China remains unclear.

The present study was therefore designed with aims to monitor the transmission of *S. japonicum* in 10 fixed surveillance sites and 30 mobile sentinel sites selected from 10 counties of Anhui, Jiangsu, Chongqing and Hubei provinces, China, which are located on the water diversion route of the projects where there is potential of transmission of schistosomiasis japonica. Our findings showed that the cases infected with *S. japonicum* were detected in 5 of the 30 mobile sentinel sites during the 5-year study period, while 27 subjects were positive for the antibody-based serum test in the 10 fixed sentinel sites; however, no infection was found. In addition, neither local nor imported livestock were found to be infected. No *O. hupensis* snails were detected in either the fixed surveillance or the mobile sentinel sites; however, the snail host was found to survive and reproduce at Chaohu Lake, inferring the potential of transmission of the disease.

The Kato-Katz technique was used to detect *S. japonicum* infection in this study. Currently, the Kato-Katz technique (three slides for a single stool specimen) is still the gold standard used for the diagnosis of schistosomiasis [6]. It has been shown that the routine Kato-Katz technique underestimates the real prevalence of *S. japonicum* in endemic areas with low-intensity infections [27]. Considering that all study areas are at a low level of *S. japonicum* infection, the missing situation of *S. japonicum*-infected villagers cannot be excluded. The search for a better diagnostic test that can be applied in the endemic field situation in China is therefore essential and should be given a high priority [28].

Recently, the elimination of schistosomiasis japonica has been put on a high agenda in China [29], and a global agenda for eliminating schistosmiasis has been set [30]. During the elimination stage when the transmission is at an extremely low level, surveillance and response becomes central to schistosomiasis control and prevention [31]. With the increase in the population migration in China, a gradual increase in the cases infected with *S. japonicum* is reported in non-endemic regions. Once the infected cases, which serve as the infectious sources of the disease, are introduced to the snail-breeding regions, there is a high likelihood of schistosomiasis transmission in the potential risk regions of China. Therefore, health education pertaining to schistosomiasis prevention and control should be strengthened in the mobile population going to endemic foci, notably the boatman and fisherman, to increase their self-protection awareness and prevent the infection [3]. Furthermore, the detection and monitoring of schistosomiasis should be strengthened in the floating populations returning from the nations or regions where schistosomiasis is endemic. 

## 5. Conclusions

*O. hupensis* is able to survive and reproduce in potential risk areas of China. It is suggested that the continuous surveillance of schistosomiasis japonica should be carried out in both the endemic foci and potential endemic regions of China, and an active, sensitive system to respond the potential risk of transmission is urgently needed. 

## References

[1] Zhou X.N., Bergquist R., Leonardo L., Yang G.J., Yang K., Sudomo M., Olveda R. (2010). Schistosomiasis japonica control and research needs. Adv. Parasitol..

[2] Wang L., Utzinger J., Zhou X.N. (2008). Schistosomiasis control: Experiences and lessons from China. Lancet.

[3] Utzinger J., Zhou X.N., Chen M.G., Bergquist R. (2005). Conquering schistosomiasis in China: The long march. Acta Trop..

[4] Yuan Y., Xu X.J., Dong H.F., Jiang M.S., Zhu H.G. (2005). Transmission control of Schistosomiasis japonica: Implementation and evaluation of different snail control interventions. Acta Trop..

[5] Ohmae H., Iwanaga Y., Nara T., Matsuda H., Yasuraoka K. (2003). Biological characteristics and control of intermediate snail host of *Schistosoma japonicum*. Parasitol Int..

[6] Zhou X.N., Guo J.G., Wu X.H., Jiang Q.W., Zheng J., Dang H., Wang X.H., Xu J., Zhu H.Q., Wu G.L. (2007). Epidemiology of schistosomiasis in the People’s Republic of China, 2004. Emerg. Infect. Dis..

[7] Wang W., Dai J.R., Liang Y.S., Huang Y.X., Coles G.C. (2009). Impact of the South-to-North Water Diversion Project on the transmission of *Schistosoma japonicum* in China. Ann. Trop. Med. Parasitol..

[8] Yang G.J., Vounatsou P., Zhou X.N., Tanner M., Utzinger J. (2005). A potential impact of climate change and water resource development on the transmission of *Schistosoma japonicum* in China. Parassitologia.

[9] Zheng J., Gu X.G., Xu Y.L., Ge J.H., Yang X.X., He C.H., Tang C., Cai K.P., Jiang Q.W., Liang Y.S. (2002). Relationship between the transmission of Schistosomiasis japonica and the construction of the Three Gorge Reservoir. Acta Trop..

[10] Zhu H.M., Xiang S., Yang K., Wu X.H., Zhou X.N. (2008). Three Gorges Dam and its impact on the potential transmission of Schistosomiasis in regions along the Yangtze River. Ecohealth.

[11] Seto E.Y., Wu W., Liu H.Y., Chen H.G., Hubbard A., Holt A., Davis G.M. (2008). Impact of changing water levels and weather on *Oncomelania hupensis* populations, the snail host of *Schistosoma japonicum*, downstream of the Three Gorges Dam. Ecohealth.

[12] Sutherst R.W. (2004). Global change and human vulnerability to vector-borne diseases. Clin. Microbiol. Rev..

[13] Zhou X.N., Yang G.J., Yang K., Wang X.H., Hong Q.B., Sun L.P., Malone J.B., Kristensen T.K., Bergquist N.R., Utzinger J. (2008). Potential impact of climate change on Schistosomiasis transmission in China. Am. J. Trop. Med. Hyg..

[14] Liang Y.S., Wang W., Li H.J., Shen X.H., Xu Y.L., Dai J.R. (2012). The South-to-North Water Diversion Project: Effect of the water diversion pattern on transmission of *Oncomelania hupensis*, the intermediate host of *Schistosoma japonicum* in China. Parasit. Vect..

[15] Cao Z.G., Wang T.P., Wu W.D., Zhang S.Q., Lv D.B., Fang G.R., Zhao F., Ling X.S., Sha J.J., Wang F.F. (2007). Potential impact of water transfer project from Yangtze River to Huaihe River on snail spread and Schistosomiasis transmission. Chin. J. Parasitol. Parasit. Dis..

[16] Zhu H., Yuan Y., Xu X.J. (2010). Risk assessment of the Water Transfer Project from the Yangtze River to Han River in middle scheme of the South-to-North Water Diversion Project on Schistosomiasis transmission and intervention measures. Chin. J. Schisto. Control.

[17] Zhou Y.B., Yang M.X., Tao P., Jiang Q.L., Zhao G.M., Wei J.G., Jiang Q.W. (2008). A longitudinal study of comparison of the Kato-Katz technique and Indirect Hemagglutination Assay (IHA) for the detection of Schistosomiasis japonica in China, 2001–2006. Acta Trop..

[18] Katz N., Chaves A., Pellegrino J. (1972). A simple device for quantitative stool thick-smear technique in *Schistosomiasis mansoni*. Rev. Inst. Med. Trop. Sao Paulo.

[19] Yu J.M., de Vlas S.J., Jiang Q.W., Gryseels B. (2007). Comparison of the Kato-Katz technique, hatching test and Indirect Hemagglutination Assay (IHA) for the diagnosis of *Schistosoma japonicum* infection in China. Parasitol. Int..

[20] Poda J.N., Sondo B., Parent G. (2003). Impact of water resource installations on the distribution of Schistosomiasis and its intermediary hosts in Burkina Faso. Sante.

[21] Ghebreyesus T.A., Witten K.H., Getachew A., Haile M., Yohannes M., Lindsay S.W., Byass P. (2002). Schistosome transmission, water-resource development and altitude in northern Ethiopia. Ann. Trop. Med. Parasitol..

[22] Sow S., de Vlas S.J., Engels D., Gryseels B. (2002). Water-related disease patterns before and after the construction of the Diama dam in northern Senegal. Ann. Trop. Med. Parasitol..

[23] Kloos H. (1985). Water resources development and Schistosomiasis ecology in the Awash Valley, Ethiopia. Soc. Sci. Med..

[24] Gryseels B., Polman K., Clerinx J., Kestens L. (2006). Human Schistosomiasis. Lancet.

[25] Steinmann P., Keiser J., Bos R., Tanner M., Utzinger J. (2006). Schistosomiasis and water resources development: Systematic review, meta-analysis, and estimates of people at risk. Lancet Infect. Dis..

[26] Yang G.J., Vounatsou P., Tanner M., Zhou X.N., Utzinger J. (2006). Remote sensing for predicting potential habitats of *Oncomelania hupensis* in Hongze, Baima and Gaoyou lakes in Jiangsu province, China. Geospat. Health.

[27] Lin D.D., Liu J.X., Liu Y.M., Hu F., Zhang Y.Y., Xu J.M., Li J.Y., Ji M.J., Bergquist R., Wu G.L. (2008). Routine Kato-Katz technique underestimates the prevalence of *Schistosoma japonicum*: A case study in an endemic area of the People’s Republic of China. Parasitol. Int..

[28] Zheng Q., Vanderslott S., Jiang B., Xu L.L., Liu C.S., Huo L.L., Duan L.P., Wu N.B., Li S.Z., Xia Z.G. (2013). Research gaps for three main tropical diseases in the People’s Republic of China. Infect. Dis. Poverty.

[29] Ross A.G., Olveda R.M., Acosta L., Harn D.A., Chy D., Li Y., Gray D.J., Gordon C.A., McManus D.P., Williams G.M. (2013). Road to the elimination of Schistosomiasis from Asia: The journey is far from over. Microbes Infect..

[30] Rollinson D., Knopp S., Levitz S., Stothard J.R., Tchuem Tchuenté L.A., Garba A., Mohammed K.A., Schur N., Person B., Colley D.G. (2013). Time to set the agenda for Schistosomiasis elimination. Acta Trop..

[31] Zhou X.N., Bergquist R., Tanner M. (2013). Elimination of tropical disease through surveillance and response. Infect. Dis. Poverty.

